# Ethical concerns when recruiting children with cancer for research: Swedish healthcare professionals’ perceptions and experiences

**DOI:** 10.1186/s12910-023-00901-4

**Published:** 2023-03-14

**Authors:** Kajsa Norbäck, Anna T. Höglund, Tove Godskesen, Sara Frygner-Holm

**Affiliations:** 1 Centre for Research Ethics & Bioethics, Department of Public Health and Caring Sciences, Box 564, SE-751 22 Uppsala, Sweden; 2 Department of Health Care Sciences, Palliative Research Centre, Marie Cederschiöld University, Box 11189, 100 61 Stockholm, Sweden; 3 Physiotherapy and behavioral medicine, Department of Women’s and Children’s Health, Box 593, 751 24 Uppsala, Sweden

**Keywords:** Paediatric oncology, Ethics, Research recruitment, Informed consent, Assent, Shared decision-making, Ethical challenges, Healthcare professionals, Qualitative research

## Abstract

**Background:**

Research is crucial to improve treatment, survival and quality of life for children with cancer. However, recruitment of children for research raises ethical challenges. The aim of this study was to explore and describe ethical values and challenges related to the recruitment of children with cancer for research, from the perspectives and experiences of healthcare professionals in the Swedish context. Another aim was to explore their perceptions of research ethics competence in recruiting children for research.

**Methods:**

An explorative qualitative study using semi-structured interviews with key informants. Seven physicians and ten nurses were interviewed. Interviews were analysed using inductive qualitative content analysis.

**Results:**

The respondents’ ethical challenges and values in recruitment mainly concerned establishing relationships and trust, meeting informational needs, acknowledging vulnerability, and balancing roles and interests. Ensuring ethical competence was raised as important, and interpersonal and communicative skills were highlighted.

**Conclusion:**

This study provides empirical insight into recruitment of children with cancer, from the perspectives of healthcare professionals. It also contributes to the understanding of recruitment as a relational process, where aspects of vulnerability, trust and relationship building are important, alongside meeting informational needs. The results provide knowledge on the complexities raised by paediatric research and underpin the importance of building research ethics competence to ensure that the rights and interests of children with cancer are protected in research.

**Supplementary Information:**

The online version contains supplementary material available at 10.1186/s12910-023-00901-4.

## Background

More than 400,000 children are diagnosed with cancer each year worldwide (henceforth “children” will refer to infants, children and adolescents from birth to 18 years of age). Improved treatments have resulted in 5-year survival rates of over 80% in high-income countries [1]. Despite progress, childhood cancer remains a major cause of death among children worldwide, and survival is still below 30% in low-income countries [1, 2]. Since children are a unique and heterogenous population, treatments and interventions need to be tailored to their needs [3]. Research will remain crucial to improve treatments, survival and quality of life for children with cancer [3–5].

Clinical trials are conducted to evaluate new treatments for children with cancer, in consecutive phases. Phase I trials are the first step in testing a new drug in humans to evaluate safety and side-effects rather than effectiveness, often in patients not responding to standard treatment. Phase II trials provide preliminary data on safety, dosage and effectiveness. Phase III trials compare the effectiveness of a drug to standard treatment, often in large randomized controlled trials [6]. In addition to clinical trials, nursing and psychosocial research is important to promote long-term psychosocial wellbeing of the growing population of childhood cancer survivors [7, 8].

Nevertheless, inviting children with cancer to research can raise ethical issues. The time following a child’s cancer diagnosis is often marked by psychological distress for the family, and medical urgency for healthcare professionals (HCPs) to start treatment [9]. Further, children and parents may find it difficult to understand information about research [9, 10]. It is ethically important to consider vulnerability in informed consent, where it can imply a lack of capacity to consent, increased susceptibility to coercion or exploitation, and increased risk of harms [11].

Ethics is a central part of the HCPs curriculum and professional competence and ethical values are incorporated into healthcare legislation and international ethical codes for healthcare and research [12–16]. HCPs are morally obliged to act in their patient’s best interest, and provide care that respects autonomy, integrity and dignity. HCPs should also contribute to medical progress through research, while protecting the welfare of the patients enrolled in medical research [16].

Children’s enrolment in research requires the parents’ permission, in addition to the child’s assent to participate [12, 16]. As surrogate decision makers for children, parents are expected to act in the child’s best interest [17]. In Sweden, children can give informed consent to research from the age of 15 if they understand the implications of participating. However, the researcher should always inform the child as far as possible, in ways appropriate to the child’s age [14]. According to the United Nations’ Convention of the Rights of the Child, which has been adopted as law in Sweden, children are entitled to active participation in decisions concerning them [18].

Decisions about a child’s care is a process involving a triad of stakeholders with possibly diverging interests and goals, which can give rise to ethical challenges [19, 20]. Research suggests that the role of children in shared-decision making (SDM) in health care is limited, with HCPs and parents mainly making the decisions [19, 21]. Children’s preferences for SDM vary, and some children prefer to take little responsibility for decisions whereas others report feeling marginalized by not being involved [21–23]. Effective patient-caregiver communication is important to promote children’s and parents’ agency and partnership in SDM [24, 25]. The role of children in SDM about research participation is less studied than SDM about care, and an evolving area of study. Insights into children’s experiences of SDM about research participation are limited but studies reporting on children’s perspectives highlight the importance of trust, supporting children’s developing autonomy, and enhancing communication with parents and HCPs [26, 27].

In paediatric oncology, research and care are often integrated. However, there are important ethical differences between care and research. In care, the goal is to benefit the child, whereas research is conducted primarily to benefit future paediatric patients through improved care [28]. Children and parents may however not distinguish research from care and assume a curative intent of research, referred to as the ‘therapeutic misconception’ [10, 28, 29]. HCPs in paediatric oncology oftentimes have dual responsibilities, providing care and conducting research [13, 30]. The dual obligations of care and research can cause role and value conflicts in recruitment [28]. Research from Swedish childhood cancer care suggests that HCPs may act paternalistically in recruitment to protect families from psychological burdens of decision-making, but thereby they may also limit their autonomy in decisions about research [31].

Identifying and monitoring ethical issues in childhood cancer research is important as science progresses [4]. Exploring ethical challenges in research recruitment from the viewpoint of HCPs is essential as HCPs play a key role in upholding ethical standards of clinical research in practice, and protecting the rights of children in recruitment. It is also important in order to promote ethical competence building among HCPs [32].

Except for Schröder Håkansson et al. (2020), studies concerning ethical challenges in recruitment of children with cancer are limited in the Swedish context. Schröder Håkansson et al. (2020) explored HCPs perspectives and focused on two ethical principles – respect for autonomy and do no harm – as well as dual role conflicts experienced by HCPs [17, 31]. In this study, we adopt a broader theoretical perspective, by incorporating ethics of care and virtue ethics. Ethics of care focus on ethical aspects of care, relationships, vulnerability and dependence between key stakeholders [33, 34]. In our view, adopting an ethics of care perspective can shed light on social and relational aspects of recruitment, including interpersonal dependency, power asymmetries and values related to caring [35]. The related concept of relational autonomy acknowledges decision-making as a relational, social and emotion-marked process and might therefore prove especially useful in paediatric settings [36, 37]. Virtue ethics focus on moral character e.g., professional values like honesty and is closely connected to the concept of ethical competence [38, 39]. Studies on HCP perceptions of ethical competence in the recruitment of children for research in a Swedish context are scarce, but HCPs in Swedish childhood cancer care have reported a need for ethical support [40–42]. Our study is an example of empirical ethics, as we combine empirical data in the form of qualitative interviews with a normative ethical analysis [43].

### Aim

The aim of this study was to explore ethical values and challenges related to the recruitment of children with cancer for research, from the perspectives and experiences of HCPs in Sweden. A secondary aim was to explore and describe HCPs’ perceptions of ethical competence in the context of recruiting children with cancer for research.

## Method

### Design

The study used a qualitative descriptive design, and followed the *Consolidated criteria for reporting qualitative research (COREQ)* [72].

### Recruitment and participants

Key informants were purposefully recruited from four childhood cancer care centres in Sweden, a professional network of nurses and via research groups at Swedish universities. Key informants are individuals who are highly knowledgeable and engaged in the topic under investigation [73]. Altogether, seven physicians and ten nurses were recruited (n = 17). All had experience from recruiting children with cancer for medical and psychosocial research. Represented in the sample are principal investigators (n = 7), clinical research nurses (n = 4), care providers assisting with recruitment (n = 1), and academic researchers conducting research involving children with cancer (n = 5). Three reported as male, thirteen as female and one as non-binary. The majority (n = 14) had over ten years’ experience of working with childhood cancer research (range 2–30 years). The mean age was 54 years (range 39–68 years). The concept of information power was used to determine sampling sufficiency. In line with this model, sample size estimation was based on the study aim, sample specificity, theoretical background, quality of dialogue and analysis strategy. Participants received study information via email, and provided written consent via email or postal letter.

### Data collection

Individual semi-structured interviews were conducted by KN during 2021, via the videoconferencing software Zoom. Interviews lasted from 40 to 52 min (mean duration: 46 min). An interview guide was developed by the authors, who have experience of clinical work within paediatrics, qualitative methods and research ethics. Participants were asked about experiences, thoughts, feelings and perspectives related to recruitment, and were encouraged to describe concrete situations/examples. The interview guide can be found in Additional files (see Additional file 1). In addition to the questions in the interview guide, prompts and follow-up questions were used. Interviews were pilot tested with one physician and one nurse, whereby one additional question was included to explore facilitating circumstances in the informed consent process. Data from the pilot interviews were included in the analysis. Interviews were audio recorded and transcribed verbatim by a professional transcriber. Prior to the interview, participants provided demographical data (e.g., age, gender, occupation, educational level, and clinical and research experience). However, the authors decided to report these selectively to maintain the confidentiality of participants.

### Data analysis

Qualitative content analysis was performed, following Graneheim and Lundman (2004) [70]. This analysis approach was chosen as it allows exploring variation and similarities in, e.g., experiences, with attention to the subject and its context [70]. As research on this topic is limited in the Swedish context, an inductive approach was adopted to keep authenticity in narratives. First, transcripts were read repeatedly by KN to become familiar with the content. Then, SFH and KN identified meaning units related to study aims for the first three transcripts and compared scope and relevance, resulting in few discrepancies. Meaning units for the remaining transcripts were identified and condensed by KN, with their central meaning preserved, and thereafter coded as close to the text as possible. KN led the analysis together with SFH, but all four co-authors discussed the coding repeatedly. Codes were compared for similarities and differences, and preliminary categories and subcategories were generated and reported, with a focus on manifest content. The analysis moved back and forth between codes, categories and transcripts. The analysis was discussed continuously among all co-authors and refined until consensus was reached for a final categorization system. First author KN has received formal training in qualitative methods and qualitative content analysis, and SFH, ATH and TG have extensive experience of qualitative research and supervising in qualitative methods. Software NVIVO 1.3 (QSR International, 2021) and Microsoft Excel were used to facilitate analysis. Examples from the analysis process are presented in Table 1.

**Table 1:** Examples from the analysis process

Meaning unit	Condensed meaning unit	Code	Subcategory	Category
*It is not sure at all that they have the same opinion as the parents… in conversations at the end of life, with dying children…there are even more different opinions.*	Children’s opinions may differ from parents and more so in end of life	Parent-child disagreement in end of life	Disagreements	Balancing roles and interests
*Many studies start already at diagnosis when the family experience chaos and I feel that the way it is now is unethical, because although each study has been ethically approved you forget that it’s too much… too much for the families…all this information in a difficult crisis situation, it’s not good.*	Families become overwhelmed by the amount of information at diagnosis	Information overload	Information burden at diagnosis	Meeting informational needs

## Results

The results describe the HCPs’ perspectives and experiences of recruiting children with cancer for research, including ethical values, challenges and ethical competence. It is important to note that recruitment was not always associated with ethical challenges for the interviewed HCPs, but was sometimes perceived as a straightforward and unproblematic process. Most HCPs in the study had long experience of working with recruiting children for research and felt confident and competent to manage ethical challenges. Nevertheless, and in line with the aim of the present study, the results will emphasize situations that the HCPs did perceive as ethically challenging. The analysis resulted in five categories: *Establishing relationships and trust, Meeting informational needs, Acknowledging vulnerability, Balancing roles and interests*, and *Ensuring ethical competence*. Several subcategories were derived (see Table 2). In the following, all categories are described and illustrated by quotes. The categories are not mutually exclusive, but sometimes overlap.

**Table 2:** Overview of categories and subcategories

Categories	Subcategories
Establishing relationships and trust	Building relationships The dilemma of trust
Meeting informational needs	Tailoring information and communication Information burden at diagnosis Difficulties due to language barriers
Acknowledging vulnerability	Parent-dependency Power asymmetries and conflicts of interests Children with poor prognosis
Balancing roles and interests	Asserting children’s rights Children’s delegation of decisions Parent’s decision authority and burden Disagreements
Ensuring ethical competence	Perceptions of ethical competence Building competence in research ethics

### Establishing relationships and trust

Many HCPs highlighted the importance of mutual trust and relationships with families, in relation to recruitment. At the same time, trust also had problematic implications for informed consent. This category comprised two subcategories: *Building relationships*, and *The dilemma of trust.*

#### Building relationships

HCPs echoed the significance of establishing a relationship with the family before inviting the child to participate in research. Relationship building, for example by chatting with the child about everyday topics, served to establish mutual trust with children and parents, and facilitated conversations about research:

*One thing that’s very important…is to establish some sort of relationship with the family, otherwise it’s not possible [to have these conversations], it’s a lot about them daring to trust you and building trust. (Nurse 1)*

The informants described that the established relationship enabled them to assess the suitability of proposing research to the family, and to identify ethical challenges:

*Having a relationship with the families and knowing that it will actually be doable, that it’s ok to ask them [about research participation]. (Nurse 8)*

However, the HCPs sometimes faced barriers to relationship building with families who had less trust, or were angry or distressed following the child’s diagnosis. Further, COVID-19 pandemic restrictions minimized the number of care contacts to reduce the risks of infection, which made relationship building more difficult, primarily for clinical research nurses without care responsibilities.

#### The dilemma of trust

HCPs described that parents, with few exceptions, had high trust in them and in healthcare in general, as well as in research. Most parents were positive to research and consented to participate in the studies they were asked about. The informants associated research positivity to the widespread societal awareness in Sweden about the importance of childhood cancer research. Some parents, more frequently foreign-born, were perceived by HCPs as having lower trust and willingness to enrol. HCPs attributed this to distrust in authorities, fears of registration and confidentiality issues, and fear of exploitation/experimentation.

HCPs were grateful for parents’ trust and committed to maintaining it. At the same time, they regarded parents’ trust as a dilemma and as a possible threat to informed consent:

*It’s also a dilemma, actually, that many families do as we say…they have very high trust in the healthcare system…the parents almost always do as we say…if you go in and just ask for a consent, you will probably get it (Physician 7)*

Hence, the interviewed HCPs were concerned about parents’ consent not always being sufficiently informed. They described that they frequently had to insist on providing information to parents who wanted to consent instantly, based on trust and despite being poorly informed. One HCP described conflicts between respecting the family’s will to abstain from information, and fulfilling the requirement for informed consent:

*I get a lot of parents telling me they can’t take any more and just want to sign…they prefer to trust us to do what’s best, but we have to get a consent and we want them to be informed. But at the same time you also have to respect them when they say, I can’t take any more, I don’t want to hear more now, but I want to consent. You cannot torment them with loads of information against their will. (Physician 4)*

### Meeting informational needs

HCPs in our study also highlighted the importance of providing tailored information, to provide a good basis for decision-making and ensuring understanding. They sometimes experienced information-related challenges, such as the family being overwhelmed by information or language barriers. This category consists of three subcategories: *Tailoring information and communication, Information burden at diagnosis* and *Difficulties due to language barriers*.

#### Tailoring information and communication

The results showed how HCPs stressed the importance of being flexible and responsive to understand and meet the informational needs of families. Addressing children with age-appropriate information was seen as a key aspect:

*We see to the whole family and we think it’s very important that the children get information as well. That we don’t exclude them… It’s really important that they are involved, because they are the ones who are sick and will go through the treatment. (Nurse 3)*

Aspects important in communication mentioned were being culturally sensitive and respectful in conversations about difficult topics. Also, the importance of body language, such as not placing oneself higher than the child, was brought up. The HCPs described how they strived for a dialogue-based and dynamic communicative approach, with an emphasis on listening and ensuring understanding, alongside providing written and verbal study information.

Further, it was highlighted that communication about research should not impede basic understanding of the child’s disease and care. The informants underscored that the difference between research and care should be clarified. However, one HCP believed that the ‘therapeutic misconception’ (i.e., failing to differentiate research from care) was not only held by parents, but also by HCPs:

*I think it often falls back on us that we do not always acknowledge the difference [between research and care] and then it’s not clear to the family, either. And I believe there are families who go through cancer treatment without having really reflected upon this. (Nurse 7)*

Moreover, the HCPs saw communicating the voluntary nature of research participation as especially important:

*For me, the most important thing has been that they understand that it’s voluntary. They participate in many different studies and it’s probably hard for them to say no. In my experience, this patient group is very grateful that so much is being done for them. (Nurse 6)*

#### Information burden at diagnosis

Recruitment for research near diagnosis was perceived as a major challenge. HCPs were concerned for the family’s wellbeing when introducing complex decision-making in a situation when they were already overwhelmed:

*They are drowned in information, and at the same time they are often emotionally blocked after getting the diagnosis. All of this is pouring over them. It’s like an avalanche of information. (Physician 7)*

The informants expressed conflicts between the value and necessity of paediatric research, and its potential burden on families:

*If treatments in paediatric oncology could advance without studies, it would of course be better, we would not have to burden them…If they could be spared these kinds of decisions, that would be great. But then again, we have our responsibility as physicians to make treatments better and safer and with improved survival. So, we must have studies. (Physician 2)*

The participants also described how they avoided or postponed recruitment if they perceived families as overburdened and unable to meaningfully engage in decision-making. However, sometimes enrolment could not wait and decisions had to be made, and at these times recruitment was particularly ethically challenging:

*What we see is that, really, they need more time…so that they are not so distressed due to their diagnosis and everything that’s happening around them. That is actually the greatest difficulty we face right now…that inclusion and sometimes also randomizations are required very early on. (Physician 7)*

It was also mentioned that legislative requirements contributed to the information burden by prescribing juridical and lengthy study information sheets, posing barriers to informed decision-making. Further, decision-making burden often increased with the number of studies the child was invited to, as well as with study complexity, uncertainty concerning the implications of participation and the start of treatment being contingent upon study enrolment. Randomized controlled studies, phase I drug trials and genetic studies were described as particularly challenging, due to study complexity and uncertainty concerning implications of participation.

Suggestions to mitigate the recruitment-at-diagnosis dilemma were mentioned by the participants. Among these were adopting a step-wise recruitment approach, repeating information throughout the research experience, implementing broader consents encompassing several similar studies and coordination of research at the organizational level, to reduce research burden on families.

#### Difficulties due to language barriers

Interviewed HCPs regarded it as their main responsibility to provide accessible and tailored high-quality information to children and parents, thereby ensuring correct understanding. Therefore, the HCPs were concerned about the quality of communication in consent processes conducted via interpreters, with non-native speaking parents and children. Moreover, they expressed frustration over the lack of translated study information sheets in many commonly spoken languages:

*If a family is of foreign origin… then, sadly, you have nothing to provide, which I think is a huge shortcoming, actually… They usually sign up, but I don’t think they always understand what they are signing up for. (Nurse 3)*

*I don’t understand why consent forms are not produced in different languages, when we are living in a multicultural country… We can’t include only people who speak Swedish or English. (Nurse 2)*

Informants were concerned about the risks of social alienation and unfair exclusion from research among non-Swedish-speaking families. They described how they did their best to inform and facilitate decision-making for these families, despite information and language barriers.

### Acknowledging vulnerability

The analysis revealed several challenging recruitment situations related to children’s and parents’ vulnerability. This category consists of three subcategories: *Parent-dependency, Power asymmetries and conflicts of interests*, and *Children with a poor prognosis*.

#### Parent-dependency

HCPs described how children’s vulnerability and need for support following a cancer diagnosis could reinforce their dependency on their parents. This dependency on parental support had implications for the informed consent process, as it could make it difficult for children to question their parents’ views and express their own:

*To feel as safe as possible, they want to be on the same side as their parents… At times, you wonder what they would think if mom and dad weren’t there. (Nurse 1)*

The informants asserted the importance of talking to primarily older children and adolescents without the parents present, to allow them to share their views without parental influence.

However, parents’ capacity to act as decision-makers was sometimes compromised by distress in the form of anger, desperation and grief following diagnosis, according to the interviewees. One HCP believed that distressed parents might be less likely to object to research proposals, or even feel a pressure to enrol:

*They are so traumatized and shocked. And then it’s easy to end up in a subordinate position because you lack the strength to resist or question things. So, they might just enrol… trying to be at ease, because they think that’s the best for their child (Physician 3)*

According to HCPs, personal psychological difficulties, intellectual disabilities, social problems and low educational background could further increase vulnerability in decision-making.

#### Power asymmetries and conflicts of interests

HCPs described ethical commitments to improve health for children with cancer through research. They also expressed voluntariness and not imposing undue influence as important values in the recruitment process. As such, some HCPs experienced role conflicts between safeguarding the child’s interests, while also acknowledging their own interests in recruitment success:

*If you are responsible for the study, you want to include as many as possible. And you also think about your reputation among colleagues; ‘he never succeeds in including anyone, but she gets everyone to enrol’. There is a lot at stake which has to do with, well... our own ambitions for research success. This creates problems all the time. (Physician 2)*

The participants problematized the fact that they knew how to communicate to increase likelihood to get consent, for example by stressing the success of research in recent decades and how it has drastically reduced childhood cancer mortality. At the same time, they were aware of the pressure and power asymmetry this created in the recruitment situation:

*I mean, if you have some experience, you could persuade anyone to enrol in a study ... And that puts you in a power position. Yes. I know how to express myself to get them to enrol... And unconsciously...and especially if it’s your own study, then you want to get everyone to enrol... No, you’re not that objective. You aspire to be, but no one is... (Physician 6)*

The interviewed HCPs experienced that the rarity of childhood cancer cases resulted in high competition in recruiting children for research, which could also contribute to conflicts of interests.

#### Children with a poor prognosis

Recruitment was described as less ethically complex if the child was relatively well in the initial stages of the disease. The informants were, however, particularly concerned about the wellbeing and rights of children with a poor prognosis or in relapse, especially in recruitment for experimental, early phase I trials. They described emotional and ethical challenges related to inviting these children to participate in research:

*These conversations are so difficult because with these families we first pull the rug from under their feet by saying their child has pontine glioma and will die and then we say, do you want to participate in a study… it’s always painful to see how immensely sad these parents are, and it can be really difficult to get through with your message without giving them false expectations. (Nurse 1)*

The interviewed HCPs stressed the importance of being honest and clear about difficult topics, and the lack of evidence for treatments offered within early experimental phase I trials. Further, they felt that the option to choose palliative care alone should be clearly communicated to families. They also highlighted the importance of managing hopes and expectations, and respecting the child’s wishes for their remaining time. The poorer the child’s prognosis, the more influence should be given to them in decisions, according to the informants. Likewise, HCPs found it inappropriate to invite children with limited chances of survival to longitudinal follow-up research, or burdening them with further medical interventions if prospects of medical gain were small.

### Balancing roles and interests

The narratives of HCPs highlighted the complexities of balancing children’s and parents’ roles and interests in decision-making. This category consists of four subcategories: *Asserting children’s rights, Children’s delegation of decisions, Parent’s decision authority and burden* and *Disagreements.*

#### Asserting children’s rights

HCPs’ perspectives on ethical values in recruitment were highly marked by notions of children’s rights and children’s perspectives. They placed children in the centre of the recruitment process and described engaging in practices with the goal of asserting children’s rights, for example through addressing children with adapted information and letting children know that their views were wanted and important. Even children too young to participate in decisions were present in conversations about research, with reference to their rights to be informed. HCPs expressed that research should be conducted on the child’s terms. Further, one HCP mentioned the importance of considering the child’s best interest from a holistic perspective, and regardless of study eligibility:

*You have to think like this, why should this child be included in this study? For whom is it good? Not just think that the child matches this study and therefore it is good for them to be involved. You need to take care of the whole child... Not all children can be in phase I trials ... and some choose not to, based on what they want with their lives. (Physician 5)*

According to the informants, children’s actual role in decision-making depended on a multitude of factors, including the child’s health status, age, maturity and personality as well as developmental factors, such as reading skills, verbal intelligence, critical thinking and potential neuropsychiatric difficulties. HCPs therefore described how they relied on a case-by-case assessment of maturity:

*You have to try to understand the degree of maturity of that particular child. It is usually not an exact age and more a matter of personality… And you need to understand the ability of that child, at that age. (Physician 6)*

HCPs also described that involvement depended on the children’s own preferences, the type of study and risks of participation. The longer disease history the child had, the more competent to make decisions they were considered, according to the informants.

#### Children’s delegation of decisions

The interviewees iterated that children tend to comply with parent’s preferences in recruitment. However, for research without medical risks, such as psychosocial intervention studies or psychometric research, children were often more involved in decisions. According to the HCPs, children and adolescents faced with the physical and emotional burdens of cancer, sometimes had little energy or interest in participating in decision-making, and instead delegated the decisions to their parents:

*Many teenagers don’t care, they don’t read these sheets. They have been taken out of school, have no friends and are hospitalized… They have been diagnosed with a life-threatening disease, so there are other things that matter more. They have been told they might die and that they will lose their hair and might become infertile. I mean, a study more or less, they leave that to their parents. I think so. Because they have enough to think about… Out of panic or because they trust their parents, you don’t really know. (Physician 2)*

#### Parent’s decision authority and burden

According to the HCPs, parents typically made decisions about enrolment on behalf of their children, especially concerning medical studies. Some HCPs meant that this was often the case, independent of the child’s age:

*I can say, yes, in most cases, the parents simply decide. Regardless of how old the child is. (Physician 2)*

The informants further stated that parents’ behaviours and attitudes impacted the children’s opportunities to participate in the decision-making process. For example, whereas some parents involved their children, others limited their participation by dominating conversations. It could be that they wanted to protect the child from difficult topics or that they assumed decision authority:

*Parents handle these situations very differently. Some are keen to include their child and ask what they think and try to explain things to the child, whereas others just decide on their own. (Physician 5)*

In such cases, the HCPs saw it as their responsibility to balance the child’s and parents’ roles in decision-making, for example by addressing the child directly in the discussions.

It was further recognized by the informants that many parents experienced burdens and fears associated with the decision-making: fears of doing wrong and causing harm to their child. The HCPs felt that parents sometimes consented mostly out of a fear of negative consequences of declining, a form of decisional regret of not having done all they could to save their child.

#### Disagreements

Most interviewed HCPs had experienced challenges related to disagreements concerning study enrolment, either between the parents or between parent(s) and the child. Disagreements were more common in cases of children being invited to early phase I and middle phase II trials. Causes of disagreements mentioned were that the parents wanted their child to try further experimental treatments, whereas the child wished to abstain, or parent altruism, i.e., that the parents wanted to benefit future children through their child’s participation, despite potential side-effects:

*At the end of life, with dying children, opinions vary more… Then the parents often think that yes, if this can help someone else, then we should do it. The altruistic view. But for their child, they cannot expect anything but side-effects. (Physician 6)*

HCPs underscored the importance of agreement between children and parents. They stated that children should not have to participate in research against their will, and were of the view that the main objective should be the child’s best and thus, research should be subordinated to care. In instances of disagreement, HCPs thought that it was better not to enrol the child in research. Being responsive to a child’s unwillingness throughout the research experience, including subtle signs, was regarded as important:

*I think we should be attentive, because there are situations when the parents want to, but the child doesn’t. Because the child doesn’t have the strength or has had enough [of research]. (Nurse 5)*

### Ensuring ethical competence

HCPs were also asked to share their perceptions of ethical competence in the context of recruitment. Unmet needs in relation to ethical support and collegial ethical dialogue were also explored. This category consists of two subcategories: *Perceptions of ethical competence* and *Building competence in research ethics*.

#### Perceptions of ethical competence

Ethical competence was described as consisting of clinical competence, scientific competence, formal ethical knowledge and ethical experience from the clinical context. Other competencies mentioned were a basic understanding of children’s cognitive development and psychological knowledge. Many HCPs relied on personal attributes, especially empathy, responsiveness and communicative skills:

*The most important thing is to be responsive and try to assess how much and what kind of information this particular family needs in this particular situation (Physician 5)*

*We often talk about informing and I think that’s a strange word. I think the greatest competence is to listen…How were my suggestions received by this family? Have they understood? (Physician 2)*

Calmness, honesty, sensitivity and self-reflexivity were also mentioned as important values and character traits. Further, the HCPs described collaboration as important, such as including both a nurse and a physician in the informed consent conversations, to ensure that no information is accidently left out. Time constraints were acknowledged as an obstacle to ethical conduct in recruitment, which is why having sufficient time was regarded as important.

#### Building competence in research ethics

Overall, HCPs felt ethically competent and confident to recruit children with cancer for studies. They did, however, highlight the importance of not becoming self-satisfied. It was acknowledged that recruitment of children with cancer for research is an ethically demanding task that requires continuous ethical reflection, dialogue and competence building.

The informants described that they had opportunities and forums to engage in collegial ethical reflection, both in formal ethical rounds and in informal discussions. The ethical climate was perceived as generally open. However, the ethical discussions mostly concerned care issues, especially acute clinical decisions, and not so often research ethics questions. As medical development is contingent on clinical research, there was a perceived need to strengthen research ethics competence, both on the personal and on the organizational level:

*Research ethics issues are not really emphasized. We have to talk about them more. Our entire treatment success depends on these kinds of studies… They are so important to us, and we can make parents agree to almost anything. And that’s problematic. (Physician 2)*

Further, HCPs perceived a need to develop competence in relation to ethical questions raised by specific types of studies, such as genetic studies and phase I drug trials.

## Discussion

The results of this study highlight the manifold ethical challenges experienced by HCPs in recruiting children with cancer for research. The informants reported ethical values and challenges related to trust, power asymmetries and vulnerability, as well as balancing the roles of parents and children in SDM. In line with Schröder Håkansson et al. (2020), HCPs in this study also reported role conflicts in balancing care with research obligations [31].

In our study, recruitment was described as a relational process, marked by dependence between the child, parents and HCPs. Trust and relationship building with the families were described as prerequisites to an ethically acceptable recruitment. Trust and relationship building are core aspects of SDM in paediatrics and form the basis for providing good care [44, 45]. Mistrust among parents was also reported by HCPs. Previous research with foreign-born parents in Swedish childhood cancer care supports that the initial trust that parents have may differ. Powerlessness, dependency, fear of discrimination and linguistic barriers can impact parents’ trust in HCPs negatively [46]. Regardless of the family’s background, parents trust may need time to develop, and must be maintained continuously by HCPs, for example through interactions demonstrating honesty, sensitivity and compassion [47, 48]. Trust and relationship building are not only of instrumental value for SDM or care provision. In ethics of care, relational values like trust are regarded as being morally valuable in themselves [34].

Trust-based consent was raised as ethically concerning and HCPs expressed tensions between fulfilling informed consent requirements and meeting parents’ wishes to abstain from information and consent based on trust. Similarly to O’Neill, we believe that a narrow focus on information can disregard the role of trust [49]. The ethical permissibility of trust-based consent has been discussed by Kongsholm and Kappel (2017), who argue that trust-based consent is ethically permissible, and compatible with autonomy, as long as there are reliable systems of ethical oversight and accountability [50]. Trust has been established empirically, as an important dimension in consent in paediatric healthcare research, and a key contributor to families deciding to enrol. Trust may however also result in children and parents underestimating risks associated to research [47]. Although HCPs are ethically obliged to support children’s and parents’ autonomy through information, children and parents have no corresponding obligation to exercise their autonomy. Trust-based consent can also be regarded an autonomous choice [17]. At the same time, parents can be argued to have ethical obligations to make informed choices to ensure that their child’s best interests are protected. The issue of trust-based consent in paediatrics is complicated and further normative and conceptual research is needed.

The interviewed HCPs strived to support autonomy in decision-making by attending to informational needs of parents and children. Like HCPs in prior studies, they relied on supportive, child-centred practices and high-quality information [25, 51, 52]. Information and communication are important to make children feel respected, safe and in control [24]. Corresponding to previous research in childhood cancer care, HCPs in this study highlighted communicative skills, empathy and honesty as important values [53]. Distress at diagnosis, and language and cultural clashes, were perceived as barriers in communication, in line with previous findings [31, 54].

Power asymmetries in recruitment created ethical challenges. Clashing with their commitment to advocate for children’s rights in recruitment, the interviewed HCPs sometimes acted in their own professional interest as researchers, striving for scientific success and reputation. Holding trust, neutrality and voluntariness as pivotal ethical standards in recruitment, the informants further described how they were worried about influencing families in the decision-making. Tensions could arise in the relationship between the parents and the child, as well as between the HCPs’ dual obligations of care and research. This could lead to conflict situations in the form of following research protocol or attending to the care needs of the patient, which is in line with the findings of Larkin et al. (2019) [55].

When considering vulnerability in recruitment, a distinction can be made between *inherent vulnerability* and *situational vulnerability*. Inherent vulnerability refers to shared human vulnerabilities, for example associated to being a child or ill. Situational vulnerabilities are situation-specific, and arise for example in informed consent processes. Assessing the complex intersections of children’s and parents’ vulnerabilities in recruitment, in a case-by-case manner, may help identify families who are particularly vulnerable [9, 56–58]. Vulnerability is not an inevitable consequence of being a child in the context of recruitment, and HCPs and parents play an important role in mitigating children’s vulnerability in recruitment by being attentive to their needs and perspectives [59].

Psychological vulnerability among children and parents following a cancer diagnosis is widely acknowledged [9, 60, 61]. For example, HCPs can have doubts about parents’ ability to safeguard children’s best interests in SDM following a child’s cancer diagnosis and at the same time being presented with a complex study protocol [57]. Decision burden and decisional regret have been reported among parents of children with cancer [62]. Parents’ vulnerability might be a significant source of vulnerability for children in SDM, given parents’ decision authority [63]. This study adds informative examples of the ethical consequences of this vulnerability in informed consent and assent processes, and highlights emotional and cognitive factors in SDM in situations of psychological and existential distress. Supporting children and parents with psychological concerns associated to SDM may be important to support autonomy [64]. Further, promoting the ability of parents to protect their children’s best interests in recruitment can make children less vulnerable [63].

The HCPs often reasoned from a children’s rights perspective and described efforts to engage children in the information and decision-making process. Despite this, they described that many children were not active in SDM, and sometimes delegated decisions to parents. Children tended to be more involved in minor decisions with lower risk, in line with previous research [25, 64]. Our results also correspond with previous qualitative research and observational studies from assent processes with children diagnosed with cancer [21, 65]. One challenge described in the results concerned balancing the roles of parents and children in SDM, especially in disagreements between parents and children, for example in phase I trials. HCPs saw it as their obligation to protect the interests of dying children, and were guided by the principle that research cannot take precedence over the patient’s best interests [16]. However, it has been argued that the meaning of that principle is unclear [66]. Especially in recruitment to phase I trials, which are not expected to benefit individual children.

Respect for individual autonomy is endorsed in biomedical ethics and emphasizes self-determination and independent choice [49]. However, individual notions of autonomy insufficiently capture the process of SDM with paediatric patients, parents and HCPs about research. Approaching autonomy in recruitment of children with cancer from a relational perspective seems more fruitful [36]. Relational autonomy is a core concept within ethics of care. It acknowledges that people are socially embedded and dependent on social relationships, and that they have interests related to these relationships. This is not necessarily a restriction of their autonomy, but rather a voluntary and emotionally preferred involvement their close ones [36].

Our results indicate that ethical challenges in the recruitment of children with cancer for research need to be understood from a broader ethical perspective than the traditional four-principle approach [17]. The emphasis on relational dimensions of research recruitment found in our study shows that the interviewed HCPs to a high degree relied on an ethics-of-care-based reasoning. Further, as also reported in a previous qualitative study, the interviewed HCPs saw sensitivity and protecting the vulnerable as central parts of ethical competence [67]. This shows that the interviewees also applied a virtue ethics perspective [39]. The emphasis on virtues is supported by previous research in which HCPs perceived ethical competence as mainly built up by moral character [68]. Further, the HCPs’ perceptions of ethical competence fit well with what Eriksson et al. (2007) described as the triad of “doing”, “being” and “knowing”. Ethical competence was described as consisting of following research-ethics standards and duties (“knowing”), possessing professional virtues and engaging in continuous ethical reflection and dialogue (“being”) and, finally, being able to act upon the ethical decisions made (“doing”) [38]. A perceived need to strengthen competence in research ethics, both on an individual and on an organizational level, was also reported, to meet the ethical standards in recruitment of children with cancer for research.

### Strengths and limitations

HCPs from four paediatric oncology centres were included, and most had long experience of recruitment, indicating good sample specificity [69]. However, a minority of HPCs were male (17.6%), and HCPs from two centres are not represented. Further, demographics are reported selectively to maintain confidentiality, which may restrict transferability, and study findings may be limited in childhood cancer care settings organized differently. Comparing nurses’ and physicians’ perspectives has not been within the scope of this study.

To maintain authenticity, an inductive, manifest analysis approach was chosen and collaborative coding was used to enrich analysis [70]. Nevertheless, qualitative analysis involve interpretation and results always represent probable meanings from a particular perspective [70, 71]. KN, who conducted the interviews, is not an HCP in paediatric oncology and has a background in psychology which may limit contextual understanding, but prevent imposed preconceptions.

## Conclusion

Ethical concerns are commonly experienced by HCPs in recruitment. This study supports that recruitment of children with cancer is a relational process, where aspects such as trust, mistrust and relationship building have to be considered, in addition to meeting informational needs. Psychological vulnerability and information burden may impact decision-making. Children’s and parents’ autonomy in SDM about research might need to be supported, not only through information but also through providing support related to psychological concerns, to reduce decision-burden. HCPs endorsed a children’s rights perspective and committed to children’s active participation in SDM. However, balancing the roles of HCPs, children and parents in recruitment can be difficult because of conflicting interests, power asymmetries and dependence. Recruiting children with cancer is an ethically demanding task that require ethical sensitivity, as well as interpersonal and communicative skills. Based on the results, building competence within research ethics is important, especially in ethically complex areas, such as genetic and phase I research. Further conceptual and normative research is needed to address the ethical concerns raised by this descriptive study, including trust-based consent, relational autonomy and situational vulnerability.

## Electronic supplementary material

Below is the link to the electronic supplementary material.

Additional File:Interview Guide

## Data Availability

The data collected in the present study are not publicly available as they contain sensitive information, but are available from the corresponding author on reasonable request.

## Abbreviations

HCPs: Healthcare professionals
SDM: Shared decision-making
