# Supplementary material for: Ethical concerns when recruiting children with cancer for research: Swedish healthcare professionals’ perceptions and experiences
Source: BMC Med Ethics. 2023 Mar 14;24:23. doi: 10.1186/s12910-023-00901-4 (PMC10012289; doi:10.1186/s12910-023-00901-4)
Supplement: Supplementary file 1 — Additional File:Interview Guide [file 12910_2023_901_MOESM1_ESM.docx]

| Additional file. Interview guide |
| --- |
| Topic I: Invitation and information |
| What do you think is particularly important when you ask and inform children with cancer and their parents about participating in studies? |
| Are there times when it feels difficult or ethically problematic to ask and inform, or when you feel unsure? |
| Are there times when, on the contrary, it feels easy and convenient to ask and inform families? |
| Topic II: Consent and assent |
| What role do parents usually play in the decision to participate? |
| How involved do children tend to be in the decision and consent process? |
| How do you see your own role in the informed consent process? |
| Have you ever felt that it would be ethically problematic to include a child in a study? |
| Topic III: Ethical competence and ethical support |
| What kind of particular competence, such as skills and abilities, knowledge or experience, do you think one needs to be able to recruit sick children in a good and ethical way? |
| Would you say that you generally feel confident that you have the competence to handle challenging situations in your work with recruiting sick children? |
| How regularly do you discuss ethical issues at your workplace? What about research ethics? Are there any obstacles to discussing ethical questions? Do you get the ethical support and guidance that you need? |
